# The effect of red grape juice and exercise, and their combination on parkinson^’^s disease in rats

**Published:** 2012

**Authors:** Fatemeh Eshraghi-Jazi, Hojjatallah Alaei, Hamid Azizi-Malekabadi, Mahin Gharavi-Naini, Aliasghar Pilehvarian, Zahra ciahmard

**Affiliations:** 1 *Department of Physiology, School of Medicine, Isfahan University of Medical Sciences, Isfahan, I. R. Iran*; 2 *Isfahan Payame Noor University, Isfahan, I. R. Iran*; 3 *Islamic Azad University, Khorasgan Branch, Isfahan, I. R. Iran*

**Keywords:** 6-OHDA, Apomorphine, Parkinson^’^s disease, Red grape juice, Rotational behavior, Treadmill running

## Abstract

**Objective: **Parkinson^'^s disease (PD) is one of the most common neurodegenerative disorders which is characterized by tremor, rigidity, bradykinesia and postural disturbances. Studies indicate that grape juice and exercise may have beneficial effects on neurodegenerative disorders. Therefore, in the present study, we evaluated the effects of red grape juice (GJ) together with treadmill running on animal model of PD.

**Materials and Methods** **: **30 male Wistar rats were divided randomly into Sham, PD, PD treated with GJ (PD-GJ), PD treated with exercise (PD-Ex), and PD treated with GJ associated with exercise (PD-GJ-Ex) groups with six rats in each. In order to obtain the PD model, 6-OHDA was infused into left substantia nigra pars compacta. In order to prove that the lesions are created and to estimate their extent, apomorphine was administered (i.p.) and total number of induced rotations was recorded during 60 minutes. Exercise was applied by treadmill and GJ was added into drinking water for 30 days and rotations test was performed again.

**Results:** Our results indicate that there was a significant difference in number of rotations between PD and Sham groups (p<0.05). At the end of experiment, number of rotations decreased significantly in both PD-GJ and PD-GJ-Ex groups (p<0.05). Exercise alone increased the number of rotations non- significantly.

**Conclusion:** Grape juice reduced rotations probably via the antioxidant agents.

## Introduction

Parkinson^'^s disease (PD) is induced by the progressive loss of neurons in substantia nigra (Van Kampen et al., 2003 ▶). The most common signs of this disease include akinesia, bradykinesia, rigidity, and tremor at rest (Howells et al., 2005 ▶). Genetic factors and several environmental toxins such as agricultural toxins and drug abuse involve in the pathogenesis of PD (George et al., 2009 ▶). Phenolic compounds as antioxidant agents which are found to be important for treatment of this disorder, have been found in vegetables, fruits, chocolates, tea, coffee, wine, grape juice, and vinegar at different levels (Davalos et al., 2005 ▶; Scalbert and Williamson, 2000 ▶). Grape juice (GJ) also contains high levels of polyphenole compounds such as flavonoids and nonflovonoids (Dani et al., 2008 ▶). GJ improves endothelial function, increases antioxidant capacity, reduces native plasma protein oxidation, and decreases platelet aggregation (Davalos et al., 2005 ▶). It has been reported that various concentrations of Concord GJ can be useful in reversing cognitive and motor deficits in aging (Shukitt-Hale et al., 2006 ▶). In addition, other studies showed physical exercise ameliorates motor performance (Yoon et al., 2007 ▶; Sunvisson et al., 1997 ▶) and raises cognitive ability in patients with PD (Yoon et al., 2007 ▶; Hurwitz, 1989 ▶). Exercise also decreases mortality and risk of PD (Yoon et al., 2007 ▶; chen et al., 2005 ▶; Kuroda et al., 1992 ▶). In another study, treadmill training ameliorated dopamine loss in hemi-parkinsonian rats (Poulton and Muir, 2005 ▶). Recently, it has been shown that exercise may be used as a therapeutic method (Yoon et al., 2007 ▶). The effect of exercise together with GJ on PD has not been reported yet. Therefore, in the present study, the effect of red GJ combined with treadmill exercise on PD was investigated in rat model. 

## Materials and Methods

**Drugs**

6-Hydroxydopamine Hydro bromide (6-OHDA HBr), Apomorphine Hydrochloride (sigma, USA), and Desipramine (parsdarou, Iran) were used in this study.

**Animals**

Thirty male Wistar rats (270-320 g) were obtained from laboratory of Animals Care and Breeding Center of Ahvaz, Jundishapur University of Medical Sciences. Rats were housed in a temperature-controlled environment with a 12h light/12h dark cycle. The experiments were done in accordance with the guidelines of Animal Ethics Committee of Isfahan University of Medical Sciences. 

**Surgery**

Animals were anaesthetized with ketamine (50 mg/kg i.p.) and xylasine (6 mg/kg i.p.) and then were placed in the stereotaxic apparatus. The skull was exposed by making a small incision on the skin. For degeneration of dopaminergic neurons, 6-OHDA solution (16 µg 6-OHDA dissolved in 4 µl of sterile 0.9% saline containing 0.2% ascorbic acid) (Sharma et al., 2007 ▶) was injected into the left substantia nigra pars compacta (SNC) at the following coordinates: 1.6 mm lateral to the midline, 4.8 mm anterior to posterior, 8.2 mm ventral to the surface of the skull (Paxinos and Watson, 1986 ▶). 6-OHDA solution was infused at the rate of 1 µl/min for 4 minutes by a 50 µl Hamilton syringe via microinjection pump. The needle remained in the place for 2 minutes after injection and then was slowly removed. Thirty minutes prior to administration of 6-OHDA, rats received desipramine (25 mg/kg i.p.) for protection of neuroadernergic projections from reuptake of 6-OHDA neurotoxin (Hritcu, 2008 ▶). The animals in Sham group were administered with an equivalent dose of vehicle (4 µl of 0.9% sterile saline containing 0.2% acid ascorbic) with the same method. 

**Behavioral test**

Two weeks after the surgery, rats were given a single injection of apomorphine (1 mg/kg i.p.) to estimate the severity of lesion (Dabbeni-Sala et al., 2001 ▶). Animals were placed individually into clear glass box (28×28×50 cm). Total number of ipsilateral and contralateral rotations towards lesion side was assessed during 60 minutes after apomorphine injection (Sahgal, 1993 ▶). Animals that made more than 100 contralateral turns/60 min were selected for the experiments (Johnston et al., 1999 ▶). Afterward, they were divided randomly into following groups with six rats in each: Sham, PD, PD treated with GJ (PD-GJ), PD treated with exercise (PD-Ex), and PD treated with GJ associated with exercise (PD-GJ-Ex) groups. Behavioral test was performed again at the end of the experiment.

**Grape juice (GJ)**

The GJ used in this study was obtained from *Vitis vinifera* grapes (variety of khoshnam) which were purchased from Baneh grape garden. Grapes were frozen and their juice extracted, manually and gradually. To prepare the 50% GJ (Shukitt-Hale et. al., 2006 ▶) red GJ and water were mixed (ratio 50:50). One day after the behavioral test, the rats in GJ groups had access to 50% GJ instead of drinking water for 30 days. Other groups had only access to drinking water.

**Treadmill running**

Rats in exercise groups were introduced to treadmill one day prior to the surgery at a speed of 17 m/min for 10 min. One day after the behavioral test and proving degeneration of SNC, rats were forced to run on the treadmill for 15 min twice daily for a total time of 30 min/day at a speed of 17 m/min for 30 days (Tillerson et al., 2003 ▶). Each running time was separated by an interval of 15 min.

**Histology**

**Figure 1:**
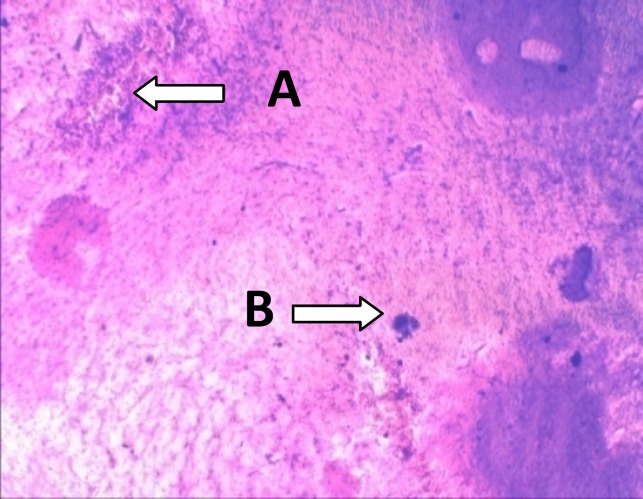
Photomicrograph scan of a coronal section (50 µm) showing the correct place of injection into SNC. A: Position of SNC; B: Line of needle in slice of the brain

At the end of all experiments and behavioral tests, rats were deeply anaesthetized by overdose of ketamine and xylasine. It was followed by perfusion of 0.9% sterile saline and 10% formalin solutions. All brains were removed and placed in 10% formalin solution. Serial sections of 50 µm thick were made with utilization a freezing microtome. Slices of the brain were stained with cresyl violet to confirm correct place of injection into SNC (Hritcu, 2008 ▶). Only data from samples with verified correct lesion site were used in statistical analysis (Figure 1). 

**Statistical analysis**

Data are presented as mean±SEM. Paired t-test was used to compare the number of rotations before and after treatment in each group. In addition, for comparing data between Sham and PD groups, independent t-test was used. The p-value less than 0.05 was accepted significant.

## Results

**Figure 2:**
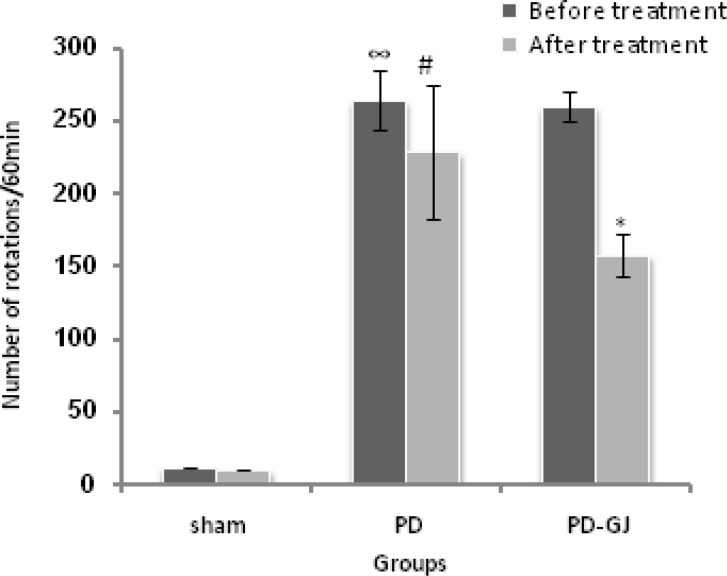
Comparing total number of rotations before and after the treatment in Sham, PD, and PD-GJ groups (n=6). Grape juice significantly decreased total number of rotations after the treatment (*p<0.05), but there were no significant differences between before and after measures in Sham and PD groups. There were significant differences between Sham and PD groups before the experiment (∞p<0.05) and also after the experiment in the number of rotations (#p<0.05). Data are presented as mean±SEM.

PD: Parkinson's disease; PD-GJ: PD treated with GJ.

**Figure 3:**
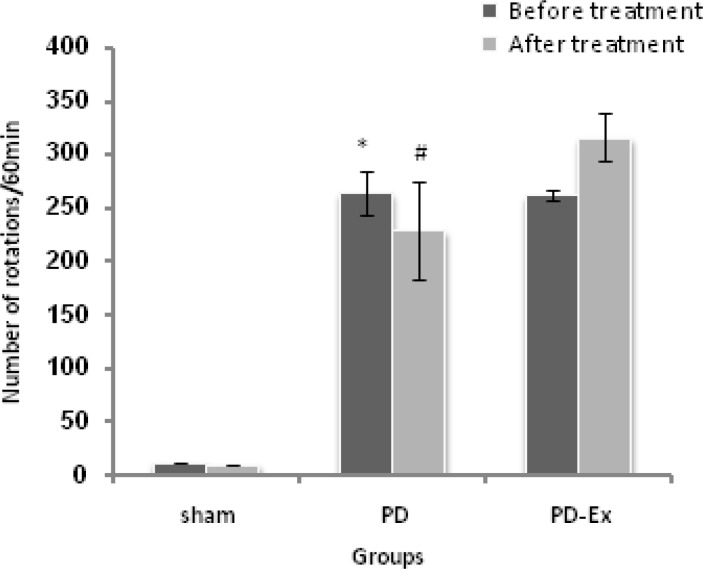
Comparing total number of rotations before and after the treatment in Sham, PD and PD-Ex groups (n=6). Exercise increased total number of rotations after the treatment but it was not significant. There were no significant differences between before and after measures in Sham and PD groups. There were significant differences between Sham and PD groups before the experiment (*p<0.05) and also after the experiment in the number of rotations (#p<0.05). Data are presented as mean±SEM.

PD: Parkinson's disease; PD-Ex: PD treated with exercise.

**Figure 4:**
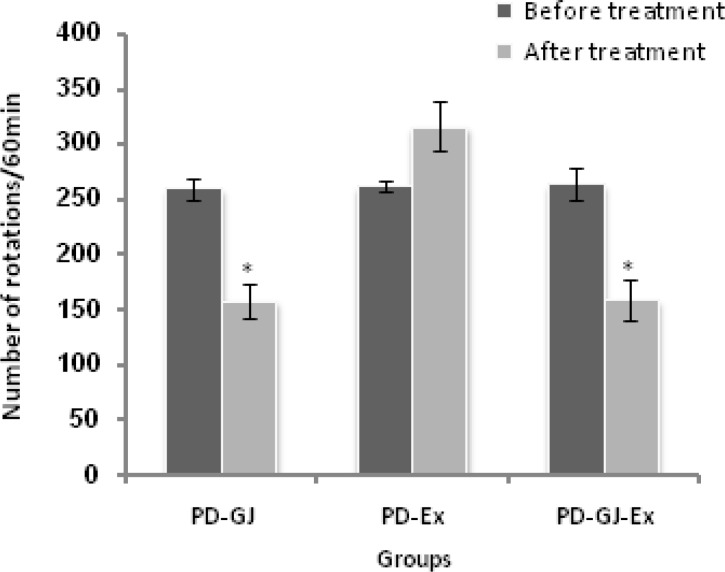
Comparing total number of rotations before and after the treatment in PD-GJ, PD-Ex and PD-GJ-Ex groups (n=6). Grape juice significantly decreased total number of rotations after the treatment (*p<0.05). Whereas exercise increased total number of rotations after the treatment, the difference was not significant. Grape juice together with exercise significantly decreased total number of rotations after the treatment (*p<0.05). Data are presented as mean±SEM.

PD-GJ: PD treated with GJ; PD-Ex: PD treated with exercise; PD-GJ-Ex: PD treated with GJ associated with exercise.

The results of the present study showed that there were no significant differences in number of rotations in Sham and PD groups before and after experiments in each group (Figures 2, 3). Statistical analyzing data from Sham and PD groups showed that apomorphine induced significant number of rotations which are resulted from degeneration of SNC dopaminergic neurons induced by 6-OHDA (p<0.05) (Figures 2, 3). After the treatment, GJ and GJ together with exercise significantly decreased numbers of rotations (p<0.05) (Figures 2, 4). 

## Discussion

In the present study, the protective effect of grape juice was demonstrated on PD. Our results showed that the rotations were decreased in PD-GJ group. In agreement with this result, another study showed that trans-resveratrol (one of the antioxidant agents in red grape) ameliorates behavioral deficits induced by 6-OHDA in Parkinson's model (Faghihi et al., 2007 ▶).

 Furthermore, it is demonstrated that green tea polyphenol attenuates behavioral abnormalities induced by apomorphine and amphetamine in hemi-parkinsonian rats (Baluchnejadmojarad and Roghani, 2006 ▶). Ahmad et al. reported that the different doses of *Ginkgo biloba* significantly decrease rotations induced by apomorphine and amphetamine in parkinsonian rats (Ahmad et al., 2005 ▶). Studies showed that *Vitis vinifera* can be useful against oxidative damage and carcinogenesis due to the presence of phenolic compounds (Dani et al., 2008 ▶; Aftab et al., 2002). Dani et al. also reported that activity of catalase (CAT) and superoxide dismutase (SOD) enzymes increase after chronic intake of purple grape juice (Dani et al., 2008 ▶). It is worth mentioning that SOD activity decreases in neurodegenerative disorders such as PD (Dani et al., 2008 ▶; Asanuma et al., 1998 ▶; Rachakonda et al., 2004 ▶). Therefore, it is possible that grape juice is able to raise activity of the enzymatic antioxidant defenses (Dani et al., 2008 ▶). In another study, Shukitt-Hale et al. reported that 50% dose of concord grape juice improves motor function in aging rats on the psychomotor tests such as rod walking, wire suspension, and small plank probably due to effects of polyphenolics on the neuronal parameters. (Shukitt-Hale et al.,2006 ▶). In this study, total number of rotations decreased after intake of red GJ, probably due to increasing activities of antioxidant enzymes and affecting neuronal parameters. Therefore, richer content of phenolic compounds and higher amount of resveratrol and anthocyanins should be able to treat PD in rats probably due to different mechanisms as mentioned above.

Our results also showed that exercise increases total number of rotations non-significantly. In agreement with this result, similar study showed that treadmill training for 30 days does not improve behavioral deficits in hemi-parkinsonian rats (Poulton and Muir, 2005 ▶). It is possible that hard akinesia in Parkinsonian animals results in limited physical activity. Therefore, reduced physical activity did not affect amelioration of parkinsonian rats. Other studies suggested that there is a reciprocal relationship between reduced physical activity and developing degeneration of dopaminergic neurons (Howells et al., 2005 ▶; Tillerson et al., 2003 ▶; Tillerson et al., 2002 ▶). In contrast to our results, it is reported that treadmill exercise reduces behavioral abnormalities in 6-OHDA-treated rats (Yoon et al., 2007 ▶; Tillerson et al., 2003 ▶) and treadmill training for 30 days decreases dopamine depletion in hemi-parkinsonian rats (Poulton and Muir, 2005 ▶). There were probably several reasons for these contrasts: First, there were differences in amount and position of 6-OHDA injection. We injected 16 µg 6-OHDA into SNC that induced degeneration of dopaminergic neurons, whereas in other studies low doses of 6-OHDA were infused into the medial forebrain or striatum (Tillerson et al., 2003 ▶; Yoon et al., 2007 ▶). 

Second, rats were forced to run at a speed of 17 m/min and this speed may be relatively high. Because of hard akinesia, animals did not coordinate with this speed. In other studies, animals were running in lower speeds and exercise could improve Parkinsonian rats (Tillerson et al., 2003 ▶; Yoon et al., 2007 ▶). 

The results of this experiment also demonstrated that treatment by red GJ together with treadmill exercise presented protective effects on parkinsonian rats similar to the PD-GJ group. Therefore, GJ plays a major role in reducing the number of rotations in parkinson^'^s model. Previously, it was reported that a combination of exercise training and vitamin E may present useful effects against age-related deficits (Asha and Ravi, 2004 ▶). Murase et Al. suggested that a combination of tea catechin and habitual exercise suppress the age-related deficits in physical performance and energy metabolism by probably improving mitochondrial function in skeletal muscle (Murase et al., 2008 ▶). However, in the current study, combination of exercise and GJ did not show to have synergic effects on reducing the number of rotations in parkinson^'^s rats. As mentioned above, it is possible that exercise could not improve GJ effects on Parkinson^'^s rats. 

## Conclusion

It is concluded that GJ reduces rotations in Parkinson^'^s rats probably through the antioxidant agents.
